# Extended lifespan in female *Drosophila melanogaster* through late-life calorie restriction

**DOI:** 10.1007/s11357-024-01233-w

**Published:** 2024-07-02

**Authors:** Michael Li, Jacob Macro, Billy J. Huggins, Kali Meadows, Dushyant Mishra, Dominique Martin, Kavitha Kannan, Blanka Rogina

**Affiliations:** 1 Department of Genetics & Genome Sciences, School of Medicine, University of Connecticut Health, Farmington, CT 06030 USA; 2 Institute for Systems Genomics, University of Connecticut Health, Farmington, CT 06030 USA

**Keywords:** Lifespan, Caloric restriction, *Drosophila melanogaster*, Aging, Life extension

## Abstract

Calorie restriction has many beneficial effects on healthspan and lifespan in a variety of species. However, how late in life application of caloric restriction can extend fly life is not clear. Here we show that late-life calorie restriction increases lifespan in female *Drosophila melanogaster* aged on a high-calorie diet. This shift results in rapid decrease in mortality rate and extends fly lifespan. In contrast, shifting female flies from a low- to a high-calorie diet leads to a rapid increase in mortality and shorter lifespan. These changes are mediated by immediate metabolic and physiological adaptations. One of such adaptation is rapid adjustment in egg production, with flies directing excess energy towards egg production when shifted to a high diet, or away from reproduction in females shifted to low-caloric diet. However, lifelong female fecundity reveals no associated fitness cost due to CR when flies are shifted to a high-calorie diet. In view of high conservation of the beneficial effects of CR on physiology and lifespan in a wide variety of organisms, including humans, our findings could provide valuable insight into CR applications that could provide health benefits later in life.

**Supplementary Information:**

The online version contains supplementary material available at 10.1007/s11357-024-01233-w.

## Introduction

Calorie restriction (CR) without malnutrition is a robust intervention known to extend lifespan across many model organisms [1–3]. This nutritional intervention has been well studied in multiple contexts, identifying countless factors that can influence an organism’s response to CR, including diet composition, caloric content, time of application, genetic background, and sex, among others [1, 2, 4–8]. The major cellular changes associated with CR include mitochondrial energy metabolism, stress response, and autophagy. Interestingly, these mechanisms are shared across several model systems including worms, flies, mice, and nonhuman primates [2, 9, 10]. There are several nutrient sensing pathways associated with CR, which are universal to different species including insulin/insulin-like growth factor 1 (IIS), AMP-activated protein kinase (AMPK), mechanistic target of rapamycin (mTOR), and the Sirtuin family [11, 12]. The Sirtuin family members (SIRT1-7) are nicotinamide adenine dinucleotide (NAD +)-dependent lysine deacetylases [13]. Overexpression of Sir2 (silent information regulator 2) increases yeast replicative lifespan, and overexpression of worm and *Drosophila* homologues extends their lifespan [14–16]. Deletion of sirtuins in yeast, worms, and flies prevents the beneficial effects of CR on physiology and longevity, although the magnitude of effect has been questioned [17–22]. In addition, SIRT1 studies have promoted search for CR mimetics such as resveratrol [11, 12, 15, 17, 18, 23]. Resveratrol has been shown to improve health and survival of mice on a high-calorie diet, improve adipose insulin signaling, and reduce the inflammation in adipose tissue of rhesus monkeys on a high-fat diet [19–21]. Importantly, human sirtuins have been atractive pharmacological targets for age-related disease, resulting in the initiation of clinical studies involving Sirt1 activators and inhibitors [3]. The apparent conservation of function between flies and humans suggests that information learned from flies may be translatable to humans. Despite this, much remains to be elucidated regarding underlying mechanisms and the potential utility of CR in extending human health.

The fruit fly *Drosophila melanogaster* has been used extensively in order to investigate CR [22]. Using the fly as a model organism, many studies have found that CR results in physical and behavioral changes, including an increase in fly spontaneous activity, decrease in female fecundity, decrease in oxidative damage, increase in mitochondrial biogenesis, activation of autophagy, increase in stress response, change in metabolism, decrease in growth, reduced age-specific mortality, and longevity extension [2, 7, 8]. Similar to rodent studies, the effects of CR in flies depend on fly sex, genetic background, calories in diet, time of diet application, and content of the diet [1, 22, 24, 25]. While genetic background influences the range of responses to CR, and its effectiveness in lifespan extension, longevity extension was reported in flies with different genetic background such as in flies collected in Dahomey [26], Germany, Dahomey, Netherlands, France, and Greece [27]. These reports examined effects of diets ranging from 0.1N to 2.0N on fly lifespan and showed that flies live longest on 0.5N diet. We followed the same protocol to make diets in our studies [26]. CR also extends life span of laboratory generated *w*^*1118*^ strain [28]. The effect of genetic background on dietary restriction–related lifespan extension was observed in the 161 Drosophila Genetic Reference Panel fly strains, in which dietary restriction in general extended lifespan [8]. Specifically, longevity extension was observed in 83% of lines examined [8]. Interestingly, dietary restriction did not extend lifespan of already long-lived Drosophila Genetic Reference Panel fly strains, possibly due to the maximum of longevity extension in those lines [8].

Examining a range of CR diets on fly physiology confirmed that CR extends lifespan in both male and female flies, but more in females than in males [22]. Dietary content, namely the yeast:carbohydrate ratio, appears to have a strong effect on the impact of dietary restriction (DR). Flies subjected to an excess of either yeast or dietary sugar have decreased longevity [29]. Good and Tatar showed that lack of yeast in diet leads to increased fly mortality and switching to a full diet increases female fecundity and reduces mortality rates to those observed in females on a constant yeast diet [30]. Chapman and Partridge showed that increasing yeast content of diet increases life-long fecundity but the highest yeast content decreases female lifespan [26].

One key question that is essential to address in regard to CR utility is whether organisms can still benefit from CR when applied late in life. Studies in mice have examined effects of transferring mice to CR at different times in life and led to different conclusions [31–34]. Extended mean and maximum lifespan and reduced incidence of tumors as cause of death were observed in mice that were subjected to CR at 19 months of age [34]. The same study showed that transferring mice from CR to a control diet at 19 months returned their rate of aging similar to controls within 8 weeks. Unlike this study, Hahn et al. reported that transferring mice from an ad libitum (AL) diet to dietary restriction (DR) at 24 months of age led to only a weak and gradual increase in survival, while transfer from DR to AL leads to an increase in mortality [31]. In a different approach, CR mice transferred to a mild fat diet at 24 months of age increased their weight and hepatic steatosis, and had a liver transcriptional profile similar to mice kept on a mild fat diet from birth, but CR appeared to protect these mice from hepatic fibrosis or change in CR-improved survival within 4 months following transfer [33]. Collectively, these studies reveal different outcomes when CR is administered late in mice life, demonstrate the importance of caloric content in diet and the genetic background of the mice, but also reveal the need to further investigate CR interventions to conclusively determine how CR impacts aging when applied late in life [35]. The short lifespan of *Drosophila* provides an advantage to address that question.

Previous work has demonstrated that flies can benefit from switching to a low-calorie diet from either a standard diet or a high-calorie diet, resulting in an immediate shift in mortality rate, gene expression profile, and lifespan extension despite previously aging on a rich diet [36–40]. Mair et al. showed that transferring female flies from a standard diet to diet containing 35% less sugar and yeast, at ages 14 or 22 days, reduces fly age-specific mortality and increases both mean and maximum lifespan [41]. Another group reported that mortality rate rapidly changed to more closely mimic mortality rate in flies subjected to a continuously restricted diet following a switch from a 40-day normal diet to DR. They revealed downregulation of genes involved in carbohydrate and fatty acid metabolism in heads and thoraces of female flies during the first 72 h following a switch [42]. However, the remaining question is how late in life female flies can benefit from shifting to CR.

Another unanswered question is the effect of switching flies from CR to full diet. Mair et al. found that switching female flies from a DR to full diet at 14 or 22 days leads to shorter mean and maximal survivorship [41]. In one study, the risk of dying after transfer to a full diet was even higher than that of flies on a lifelong, high-calorie diet [43]. In this particular study, the authors also reported that restricted flies were less fecund when returned to a full diet [43]. This finding suggested that there may be a hidden cost associated with CR, challenging the view that restriction is a stimulus that cues an organism to invest in somatic maintenance. However, the hypothesis that CR imposes a hidden cost was later contested by data in an outbred population of female flies, showing that CR did not result in a fitness cost that hindered fecundity upon refeeding [44]. Such differences in experimental results could be due to various reasons, including differing experimental designs and/or genetic background, as both can influence response to CR [45].

Our study differs from reported studies in diet composition, timing of diet shift, and examination on female physiology. Here, we examined if wild-type, female *Canton-S* (*CS*) flies could benefit from application of CR late in life. We also examined the effects of shifting diets with different caloric content on fly metabolic and physiological adaptation. Rather than shifting flies from standard to restricted diets, as has been done previously, we shifted flies from a high- to a low-calorie diet (and vice versa) in order to increase clinical relevance. We reasoned that since most humans with metabolic disorders eat above the daily recommended allowance, shifting flies to and from a high-calorie diet would better recapitulate human response to CR [37]. Several human randomized clinical trials provided evidence that CR, or time-restricted feeding (TRF), corrects metabolic dysfunction and leads to a reduction in adiposity and weight loss especially in obese individuals, individuals with type 2 diabetes, or individuals with metabolic syndromes [2, 3, 13, 38, 39]. However, these trials mainly include middle age individuals [38, 40], necessitating a deeper understanding of how diet interventions impact older individuals. Due to the increase in age-associated incidence of metabolic syndrome, maintaining a better understanding of age-dependent application of CR is vital.

## Methods

### Fly strains, maintenance, and diet

We used the wild-type *Canton-S (CS)* line obtained from the Bloomington Stock Center (Stock number 1). *CS* flies were reared on food containing 25 mg/mL tetracycline for three generations to eliminate *Wolbachia*. This treatment was followed by growing flies for at least 10 generations in tetracycline-free food. Standard laboratory corn media was used to grow *CS* fly Parental (P) and F1 from which cross was set to for F2 (experimental) fly collections [23]. Each cross for P, F1, and F2 (experimental) was done by crossing 10 virgin female and 9 male flies who were 4–10 days old. The flies were passed to a new vial with corn media every 2 days. The time each group of flies stayed in one vial starting with P flies was the same to avoid any effects of larval density on longevity. F2 progeny was collected every 24 h. Each vial contained 50 flies—25 males and 25 females. Flies were kept on a low (L) (50 flies per vial), or on a high (H) (50 flies per vial) diet. The total number of flies in each experiment is listed in Tables 1 and 2, and Supplemental Tables 1 and 2 Flies were maintained in a humidified temperature-controlled environmental chamber at 25 °C (Percival Scientific) on a 12-h light:dark cycle with light on at 6:00 AM. Standard laboratory corn diet was used for setting the crosses, while experimental diet is marked as low L = 0.5N and high H = 3.0N [23].

**Table 1:** Effects of shifting *Canton-S* female flies from a high (H)- to a low (L)-calorie diet on longevity compared to longevity of flies on lifelong high-calorie diet

Food	Time on L diet	*N* (*n* censored)	Mean LS (% change)	*X*^2^	*p*	Maximal LS (% change)
H	Lifelong	402 (12)	46.7 (− 91.2)	751.9572	< 0.0001*	68.2 (− 62.2)
L	Lifelong	439 (6)	89.3			110.6
H	Lifelong	402 (12)	46.7 (− 84.2)	423.5131	< 0.0001*	68.2 (− 53.9)
HLD20	D20	207 (4)	86.0			105
H	Lifelong	402 (12)	46.7 (− 26)	47.8706	< 0.0001*	68.2 (− 59)
HLD50	D50	217 (7)	59.1			108.2
H	Lifelong	402 (12)	46.7 (− 13.9)	16.5431	0.0742	68.2 (− 53.8)
HLD60	D60	211 (10)	53.2			104.9

*L* low-calorie diet, *H* high-calorie diet, *HLD20* flies shifted from a high- to a low-calorie diet at day 20, *HLD50* flies shifted from a high- to a low-calorie diet at day 50, *HLD60* flies shifted from a high to low-calorie diet at day 60

*N* = Number of flies in experiment used for calculating mean and maximal lifespan

*n censored* = number of flies that died between 0 and 10 days, and are not included in listed *N* and are not included in calculation of mean and maximal lifespan. The total number of flies in experiments on day 0 is *N* plus *n* flies)

^*^Statistically significant

**Table 2:** Effects of shifting *Canton-S* female flies from a low (L)- to a high (H)-calorie diet on longevity compared to longevity of flies on lifelong low-calorie diet

Food	Time on L diet	*N* (*n* censored)	Mean LS (% change)	*X*^2^	*p*	Maximal LS (% change)
L	Lifelong	439 (6)	89.3 (− 48)	751.9572	< 0.0001*	110.6 (− 38)
H	Lifelong	402 (12)	46.7			68.2
L	Lifelong	439 (6)	89.3 (− 44)	565.7714	< 0.0001*	110.6 (− 31)
LHD20	D20	207 (6)	49.8			76.3
L	Lifelong	439 (6)	89.3 (− 35)	526.5062	< 0.0001*	110.6 (− 30)
LHD50	D50	226 (4)	58.0			77.9
L	Lifelong	439 (6)	89.3 (− 31)	454.1635	< 0.0001*	110.6 (− 26)
LHD60	D60	218 (6)	62.0			82.2

*L* low-calorie diet, *H* high-calorie diet, *LHD20* flies shifted from a low- to high-calorie diet at day 20, *LHD50* flies shifted from a low- to a high-calorie diet at day 50, *LHD60* flies shifted from a low- to a high-calorie diet at day 60, *N* number of flies in experiments used for calculating mean and maximal lifespan, *n censored* number of flies that died between 0 and 10 days, and are not included in listed *N* and are not included in calculation of mean and maximal lifespan. The total number of flies in experiments on day 0 is *N* plus *n* flies)

^*^Statistically significant

Standard laboratory corn diet (1L) was prepared by mixing 113 g sucrose (MP Biomedicals, Inc, #ICN90471380) and 28 g brewer’s yeast (MP Biomedicals, Inc, #ICN90331280) in 643 mL water. The food mixture was stirred on an electric plate stirrer (Thermo Scientific Cimarec Stirring Hot Plate) at room temperature for 10 min and then autoclaved for 20 min. Forty-nine grams of cornmeal (LabScientific FLY800910, extra fine mesh), and 8.1 g agar (SciMart LLC, DR-820-25F) were mixed in 268 mL water and added to autoclaved food mixture and autoclaved again for 20 min. The food mixture was stirred on an electric plate stirrer until it cooled down to 65 °C. When the food was cooled down to 65 °C, 2.4 g tegosept (Methyl4-hydroxybenzoate, Sigma # H5501) dissolved in 10.7 mL 100% ethanol was added. The fly food dispenser (Droso-Filler, Genesee Scientific) was used to dispense food into wide shell vials (Fisher Scientific #AS519). The vials were covered with large Kimwipes and cheese cloth. After food was cooled down, the vials were covered with BuzzPlugs (Fisher Scientific #AS275) and kept at 4 °C cold room. The vials were warmed to room temperature before use.

#### Experimental diets

The two experimental caloric diets are standardized based on 1.0N diet, which has 100 g/L of sucrose (MP Biomedicals, Inc, #ICN90471380), 100 g/L of brewer’s yeast (MP Biomedicals, Inc, #ICN90331280), and 20 g/L of agar (SciMart LLC, DR-820-25F). The diet also has 2.3 g tegosept (Methyl4-hydroxybenzoate, Sigma # H5501) dissolved in 10 ml 100% EtOH [22, 23, 26], which is added after the diet was cooled down to 65 °C, following autoclaving.

#### Preparation of 0.5N, low-calorie (L) diet

Water was poured into a large stainless-steel pot placed on an electric plate stirrer and stirred. Slowly, 50 g/L of sucrose, 50 g/L of brewer’s yeast, and 20 g/L of agar were added to the water and mixed on the electric plate stirrer at room temperature for 10 min. The food was then autoclaved for 20 min. The food mixture was cooled down to 65 °C, while mixed on an electric plate stirrer. Once the food was cooled down, 2.3 g tegosept dissolved in 10 mL 100% EtOH was added to the food mixture. The fly food dispenser was used to dispense food into wide shell vials.

#### Preparation of 3.0N, high-calorie (H) diet

Water was poured into large stainless-steel pot placed on an electric plate stirrer and started to stir. Slowly 300 g/L of sucrose, 300 g/L of brewer’s yeast, and 20 g/L of agar were added while constantly stirring. The ingredients were stirred at room temperature for 10 more min and then autoclaved for 20 min. The food mixture was let cooled down to 65 °C while constantly stirred on an electric plate stirrer, and then 2.3 g tegosept dissolved in 10 mL 100% EtOH was added to food mixture. The fly food dispenser was used to dispense food into wide shell vials.

### Lifespan studies

Lifespan studies were performed using 10–20 vials per experimental condition, with each vial containing 25 male and 25 female flies (50 flies total). Male and female flies were collected within 24 h following eclosion and maintained in plastic vials containing high- (H) or low-calorie (L) diet, and maintained in a humidified temperature-controlled environmental chamber at 25 °C (Percival Scientific) on a 12-h light:dark cycle with light on at 6:00 AM. While males were aged together with female flies, here we present data for only female flies, while male findings are presented elsewhere [46]. The number of flies in each survivorship study is listed in Tables 1 and 2 and Supplemental Tables 1 and 2. Longevity data were censored for early mortality (1–10 days) to remove death due to post-eclosion maturation, or other deaths that are not related to aging. The number of censored flies is listed in brackets in Tables 1 and 2, and Supplemental Tables 1 and 2.

Two different shifting experiments were performed. In experiment 1, two groups of *CS* flies were aged on a high-calorie (H) or low-calorie (L) diet for their whole lifespan. An additional three groups of flies aged on L, and three groups aged on H diet, were shifted to opposite food at ages 20, 50, or 60 days. Flies were passed every day from day 1 and the number of dead flies were counted. There were between 10 and 20 vials per shifting experiment. The total number of flies in each shifting performed in experiment 1 is provided in Tables 1 and 2.

In experiment 2, two groups of *CS* flies were aged their whole lifespan on a H or L diets, and five additional groups that began on H from birth and were moved to L diet at either 10, 20, 30, 40, or 50 days. Five additional groups of flies were kept on L diet from birth and shifted from L to H diet at either 10, 20, 30, 40, or 50 days. They were passed every 2 days up to age 10 days; after 10 days, they were passed daily and the number of dead flies were counted. There were between 10 and 12 vials per shifting experiment. The total number of flies in each shifting performed in experiment 2 is provided in Supplemental Tables 1 and 2.

### Fecundity

Life-long fecundity was determined by performing daily counts of eggs laid by an individual female fly placed with one male fly and averaging across lifespan; average life-long fecundity for an experimental condition was calculated by averaging fecundity across replicate vials. There were 20 replicate vials of the *CS* flies, each vial with one female and male pair in each of the following experiments: two groups of flies were kept on L or H calorie diet the whole lifespan, another two groups of flies were shifted from L to H or H to L diets at 10 days of age, and two groups were shifted from L to H or H to L at 50 days of age. Every day, each pair of a male and a female fly were passed into new vials, and the number of eggs present in each vial was determined by counting the eggs on the microscope [47]. The number of eggs is listed in Table 3.

**Table 3:** Effects of shifting *Canton-S* female flies from a high- to a low- or from a low- to a high-calorie diet on female lifelong fecundity and during 5 day periods before and after shifting

Diet	Time on diet	Lifelong *N* eggs	SEM	6-10D	SEM	11-15D	SEM
H	Lifelong	906.5	79.0	221	19.2	206	19.8
HLD10	0–10 H, 10-rest L	884.80	68.9	243	23.2	114	9.0
LHD10	0–10 L, 10-rest H	668.65	86.2	70	4.8	172	21.1
L	Lifelong	593.4	31.6	58	3.2	77	2.0

Average number of egg/fly for *Canton-S* female flies kept on a high (H)- or a low (L)-calorie diet lifelong, or shifted from a H to a L, or L to H at day 10. The number of average eggs/fly listed are laid during female lifetime, during 6–10, or 11–15 day periods. Each *CS* females was placed along with one male flies per vial on day 0. SEM = of the average eggs laid during listed periods. There were 20 females in each experiment. *N* = number of eggs

*L* low-calorie diet, *H* high-calorie diet, *HLD10* flies shifted from a high- to a low-calorie diet at day 10, *LHD10* flies shifted from a low- to a high-calorie diet at day 10

### Biochemistry

*Canton-S (CS)* flies were collected and aged as described above on L or H diets. At ages 20 and 50 days, subgroups of flies were transferred to the opposite diet. Analysis was done on flies aged their whole life on L or H diet at age 20 or 50 days. Groups of flies transferred to the opposite diet at 20 days were used for analysis at ages 21, 22, and 25 days. Similarly, flies that were transferred at 50 days to the opposite diet were used for analysis at ages 51, 52, and 55 days. Flies were sorted on CO_2_ by sex. Three biological replicates of 10 female flies per replicate were anesthetized on CO_2_, weighed, and homogenized in 100 μl of cold homogenization buffer (0.01 M KH_2_PO_4_, 1 mM EDTA pH 7.4) with hand-held motorized pestle-homogenator for 1 min. An additional 900 μl of homogenization buffer was added and the tube was spun down at 2000 rpm for 2 min at 4 °C. Twenty-five microliters of each homogenate was aliquoted into three wells of each of six 96-well plates. The plates were kept on dry ice allowing homogenates to be frozen immediately and kept at − 80 °C until quantification. Before quantification, the plates were left to warm up to room temperature for 15 min. For glucose, peroxidase-glucose oxidase (PGO) enzyme plus color reagent was added to each well (Glucose Assay Kit: Sigma GAG020, PGO Sigma P7119), the plate was incubated at 37 °C, and the optic density was read at 450 nm using a Tecan Spark Microplate Reader. For glycogen, the procedure was the same as glucose except amyloglucosidase (Amyloglucosidase from *Aspergillus niger*, Sigma 10,115) was added to each well in addition to the other enzyme. For trehalose, the procedure was the same as glucose except the samples were incubated with trehalase at 37 °C before adding PGO (Trehalase from porcine kidney, Sigma T8778). Protein was determined using Total Protein Kit, Micro Lowry, Peterson’s Modification (Sigma TP0300). The plate was incubated at room temperature and was read at 750 nm. The triglycerides were determined enzymatically using Serum Triglyceride Determination Kit (Sigma TR0100; Glycerol Standard Solution Sigma G7793) [48].

### Statistical analysis

Egg production data from the periods between 6–10 and 11–15 days were analyzed separately using the Kruskal–Wallis test (Table 3), with post hoc analysis conducted using Dunn’s test, both performed with GraphPad Prism 9.4.1. Results represent SEM. There were 20 female flies for each experimental condition. *p*: 0.05 (*), 0.01 (**), 0.001 (***), < 0.0001 (****).

Lifelong egg production data were analyzed using the Kruskal–Wallis test. Post hoc testing was performed using Dunn’s multiple comparison test. A table with the results of the post hoc test with the *p*-values for each comparison that was conducted is in Table 4.

**Table 4:** Effects of shifting *Canton-S* female flies from a high- to a low- or from a low- to a high-calorie diet on lifelong fecundity

Dunn’s multiple comparisons test	Mean rank diff	Summary	Adjusted *p* value
H vs. HLD10	2.200	ns	> 0.9999
H vs. LHD10	17.75	ns	0.0943
H vs. L	25.85	**	< 0.01
HLD10 vs. LHD10	15.55	ns	0.2060
HLD10 vs. L	23.65	**	< 0.01
LHD10 vs. L	8.100	ns	> 0.9999

Post hoc results of analysis of lifelong egg production presented in Fig. 3A

*L* low-calorie diet, *H* high-calorie diet, *HLD10* flies shifted from a high- to a low-calorie diet at day 10, *LHD10* flies shifted from a low- to a high-calorie diet at day 10

^*^Statistically significant

^**^Statistically significant

Biochemistry data from 20- and 50-day-old flies were analyzed separately using two-way ANOVA, with post hoc analysis conducted using Tukey’s test, both performed with GraphPad Prism 9.4.1. Results represent means ± SE of three biological replicates containing ten flies per replicate. Final data are expressed per individual fly. *p*: 0.033 (*), 0.002 (**), 0.0002 (***), < 0.0001 (****). Longevity data were censored for early mortality (1–10 days) and analyzed by log-rank tests using the JMP16 program. The number of censored flies is listed in brackets in Tables 1 and 2, and Supplemental Tables 1 and 2.

### Hazard ratio

Survivorships’ data were preprocessed using custom python scripts and binned in groups of 10 days. The hazard ratios were calculated on binned data from female flies, which were shifted from H to L or L to H diet at 20, 50, and 60 days and compared to flies which were kept on constant H or L diets. Custom scripts in R (Version: 4.1.2) were written to calculate hazard ratios using the cox proportional hazard regression model. The model was made, and hazard ratios calculated using the survival library (Version: 3.2–13). We used the cox model tabcoxph function from the tab library (Version: 5.1.1) to generate a summary table. To check for the proportional hazard assumption for a cox regression model, a scaled Schoenfeld residual test was performed and visualized for the covariates using the R libraries survival and survminer (Version:0.4.9) respectively. Data are presented in Supplemental Table 3A-C.

**Table:** 

Variable	Variable name
Beta (SE)	Coefficient (standard error)
HR (95% CI)	Hazard ratio (confidence intervals)
*p*	*p* values

HR is calculated from Beta by taking its exponent value. Values of Beta are determined by the cox regression model. Log-rank test was used to calculate the *p* values

HR = 1: no effect, HR > 1: increase in hazard, HR < 1: decrease in hazard

## Results

### Calorie restriction increases lifespan of female flies when applied late in life

**Fig. 1:**
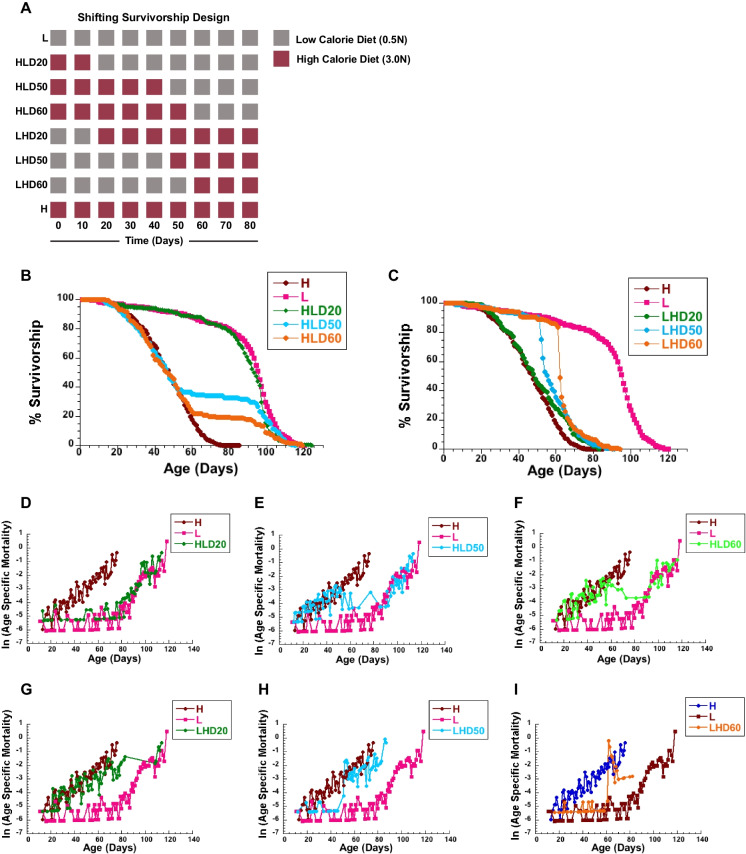
Shifting female flies to diet with different caloric content effects lifespan and mortality.** A** Schematic diagram of the experimental design: a group of female flies were aged on a low-calorie (L), or a high-calorie diet (H). Additional three groups of flies were shifted from H to L diet at day 20 (HLD20), 50 (HLD50), or 60 (HLD60), and another three from L to H diet on day 20 (LHD20), 50 (LHD50), or 60 (LHD60). **B**–**I** Survivorships (**B**, **C**) and mortality rates (**D**–**I**) of female flies shifted from H to L at day 20 (HLD20), day 50 (HDL50), or day 60 (HLD60) (**B**, **D**–**F**) or from L to H diet on day 20 (LHD20), day 50 (LHD50), or day 60 (LHD60) (**C**, **G**–**I**). Number of flies: L = 439, H = 402, HLD20 = 207, HLD50 = 217, HLD60 = 211, LHD20 = 207, LHD50 = 226, LHD60 = 218. Survivorship curves and mortality rate were analyzed by long-rank test JMP16 program

**Fig. 2:**
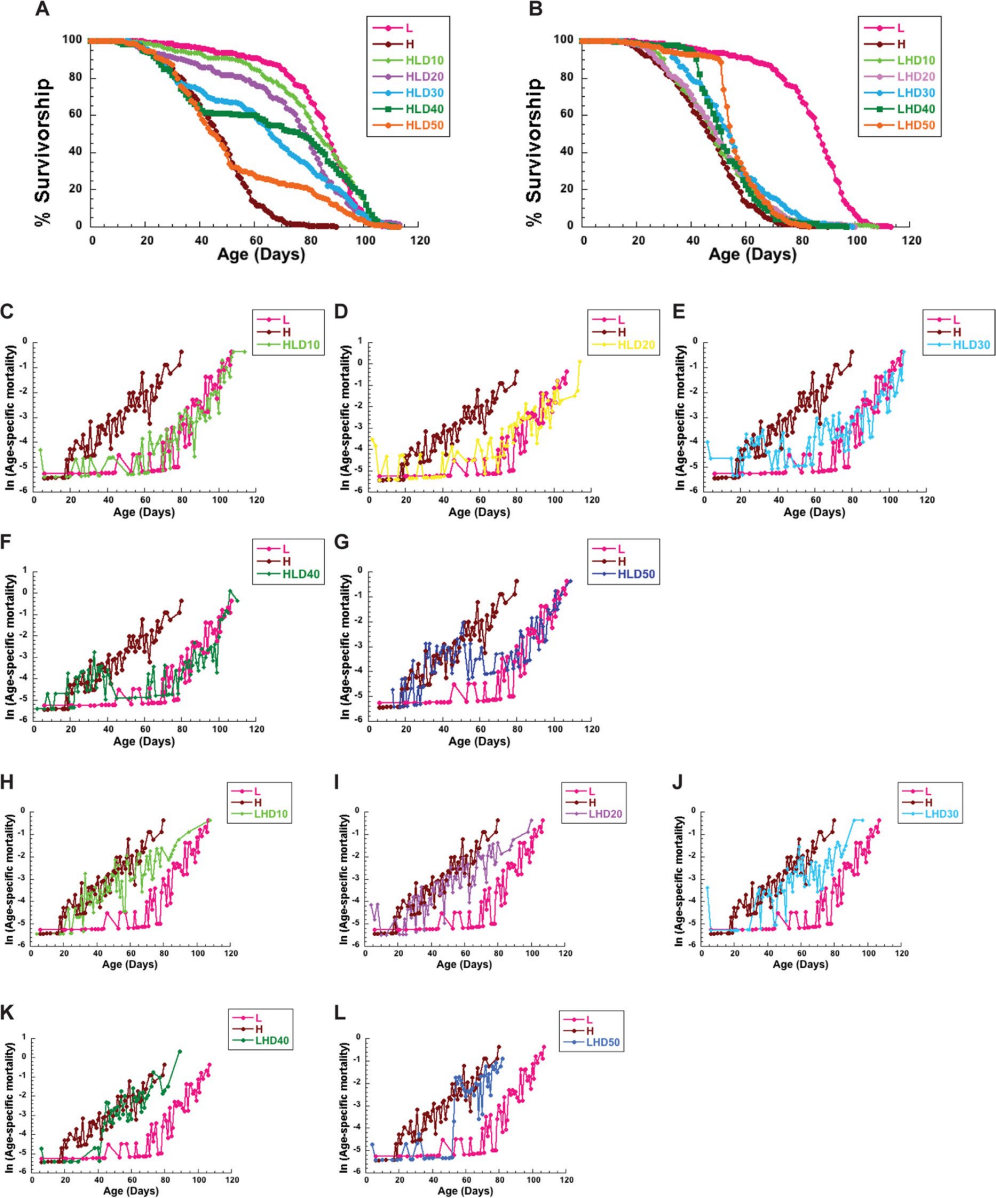
Shifting diets at different times during aging has immediate effects on female lifespan and mortality. Survivorships (**A**, **B**) and mortality rates (**C**–**L**) of female flies shifted from a high (H)- to a low (L)-calorie diet at day 10 (HLD10), day 20 (HLD20), day 30 (HLD30), day 40 (HLD40) or day 50 (HDL50) (**C**–**G**) or from L to H diet on day 10 (LHD10), day 20 (LHD20), day 30 (LHD30), day 40 (LHD40) or day 50 (LHD50) (H–L). Number of flies: L = 194, H = 227, HLD10 = 219, HLD20 = 225, HLD30 = 211, HLD40 = 222, HLD50 = 229, LHD10 = 231, LHD20 = 242, LHD30 = 199, LHD40 = 228, LHD50 = 224. Survivorships curves and mortality rate were analyzed by log-rank test JMP16 program

In order to probe how late in life *CS* female flies aged on a high-calorie diet can benefit from CR, we performed a series of survivorship studies in which flies were shifted from a high- to a low-calorie diet at various time points. In our initial survivorship studies, flies were shifted at days 10, 50, and 60, as indicated in Fig. 1A. As expected, flies raised on a low-calorie diet lived significantly longer than those raised on a high-calorie diet (Fig. 1B, Table 1, *p* < 0.0001). Strikingly, flies shifted from a high- to a low-calorie diet on days 20 and 50 displayed significant lifespan extension (Fig. 1B, Table 1, *p* < 0.0001). When flies were shifted to a low-calorie diet at the age of 50 days, the response was dramatic, with a 59% increase in maximal lifespan compared to flies on a lifelong, high-calorie diet (Table 1). When shifted at day 60, lifespan was not significantly different from flies on a lifelong, high-calorie diet (Fig. 1B, Table 1, *p* = 0.07), but the remaining, old flies still responded to a low-calorie diet with a 53.8% increase in maximal lifespan. Both day 50 and day 60 shifts lead to dramatic changes in survivorship, which becomes almost flat after the shift and then follows a similar trajectory of survivorship of flies on a low-calorie diet at about 100 days of age (Supplemental Fig. 1A,B). In a second set of experiments performed, an immediate and profound increase in survivorship was also observed when flies were shifted from a high to a low diet starting at 10 days of age all the way to 50 days of age (Fig. 2A). Again, flies shifted to a low-calorie diet gained a significant increase in survivorship compared to flies on high-calorie diet (Fig. 2A, Supplemental Tables 1, 2). *Together, these data suggest that female flies aged on a high-calorie diet can benefit from CR, even when implemented at an old age.*

To further examine the effects of late-life caloric shifting on female flies, we also performed opposite experiments in which flies were started on a low-calorie diet, and then shifted to a high-calorie diet at days 10, 50, and 60, as indicated in Fig. 1A. In contrast, shifting from a low- to a high-calorie diet dramatically shortened female lifespan (Fig. 1C, Table 2, *p* < 0.0001). Interestingly, the shortening in lifespan was related to the age of the flies when shifted, with flies shifted at 60 days exhibiting the smallest, negative effects on lifespan. For example, when shifted to a high-calorie diet at day 20, there was a 44% decrease in median lifespan compared to flies on a lifelong, low-calorie diet (Table 2). However, when shifted at day 60, there was a 31% decrease in median lifespan between shifted flies and those on a lifelong, low-calorie diet (Table 2). *Together, our data suggests that a high-calorie diet is detrimental to fly lifespan at all ages, despite previous exposure to a low-calorie diet, but that lifespan is immediately responsive to caloric shifts.*

### Calorie restriction alters age-specific mortality

We calculated age-specific mortality rates for comparisons of death vulnerability at different ages to provide insight into the reasons for a given intervention’s effect [41]. In this case, we were interested in the effects of age and caloric content on mortality risk in female flies. We first calculated age-specific mortality rates for flies that underwent shifts from a high to a low-calorie diet. Under these conditions, flies responded with an immediate decrease in risk of death upon the switch, with rates quickly mirroring flies raised on a lifelong, low-calorie diet (Fig. 1D–F).

This was true at each age tested, including 60 days, suggesting that low-calorie diets decrease risk of death even after a history of high-caloric diets. Importantly, the slope of the mortality trajectory was similar between switched flies and those on a lifelong, low-calorie diet, suggesting a decrease in short-term risk of death rather than a decrease in the accumulation of age-related damage when switched [41]. We used cox regression analysis to calculate differences in risk of dying, as estimated by hazard ratios (HRs) between flies on a constant low- or high-calorie diet (control) and low to high or high to low shifted flies (experimental) (Supplemental Table 3A-C) [31]. Shifting flies from a high- to a low-calorie diet at day 20 reduced their risk of dying, compared to flies on a high-calorie diet, indicated by a hazard ratio below 1 for every time period calculated after the shift (Supplemental Table 3A). Similar reduced risks of dying were observed in flies shifted on day 60, while in female flies shifted on day 50, reduction was observed in period between 60 and 70 days (Supplemental Table 3B,C).

Considering previous evidence that flies on a restricted diet respond negatively when switched to fully fed conditions [43, 44], we wanted to examine whether low-calorie diets at the start of life would be protective or detrimental to flies when switched to a high-calorie diet later in life. To do this, we also calculated age-specific mortality rates of flies that were shifted from a low- to a high-calorie diet. Our previous study in males indicated that flies gain an instantaneous increase in mortality risk when switched to a high-calorie diet, one that is higher than that of flies raised on a constant, high-calorie diet [46]. This was not necessarily true of females. While there was an increase in risk at all ages tested when switched, younger flies (day 20) seemed to be more resistant to the harmful effects of a high-calorie diet than older flies (days 50 and 60). At day 20, the risk of death, as estimated by hazard ratios, was not significantly different between flies raised on a high-calorie diet and those shifted from a low- to a high-calorie diet during that time (Supplemental Table 3A). At day 50, females shifted from a low- to a high-calorie diet had a higher risk of dying than flies on a high-calorie diet, but only for the first 10 days. However, as flies in this group aged, they showed a lower risk of mortality than those on a high-calorie diet, again, resulting in a longer lifespan than flies on constant, high-calorie diet (Fig. 1H and I, Table 2). To further appreciate the increase in acute risk of death that occurs after switching from a low- to a high-calorie diet at an old age, we evaluated survivorship immediately after the shift occurred (Supplemental Fig. 1A). When evaluating survivorship in this way, it became immediately obvious that survivorship decreased rapidly immediately following the shift, at a rate that was faster than flies on a lifelong, high-calorie diet, which corresponded with the increase in mortality around day 50 (Supplemental Fig. 1A). However, by day 55, survivorship then proceeded at the same rate in flies switched from low to high as those on a high-calorie diet (Supplemental Fig. 1A). This effect was further exacerbated when the flies were shifted at day 60 (Supplemental Fig. 1B). This result was even more obvious in our second survivorship study, which showed low to high shifted flies had an intermediate increase in mortality rate that was higher than flies on a low-calorie diet, but lower than those on a high-calorie diet, at days 10, 20, and 30 (Fig. 2H–J, Supplemental Tables 1, 2).

Ultimately, this transient increase in mortality may be due to the effects of age, which could render flies less effective at dealing with the negative effects associated with a high-calorie diet [46]. Similar age-associated increases in mortality were observed in female flies maintained on a standard laboratory corn diet and mated at different ages, suggesting age-associated, rather than diet-related, increases in mortality [47]. Regardless, a previous history of a low-calorie diet does not seem to be costly to young flies, and while switching may be acutely harmful to flies as they age, it does not impose a lifelong risk that is any higher than that of flies on a high-calorie diet. *Taken together, shifting from a high- to a low-calorie diet is profoundly effective, even at an old age. Shifting from a low- to a high-calorie diet shows age-dependent increases in mortality.*

### Female egg production rapidly responds to caloric shifts

**Fig. 3:**
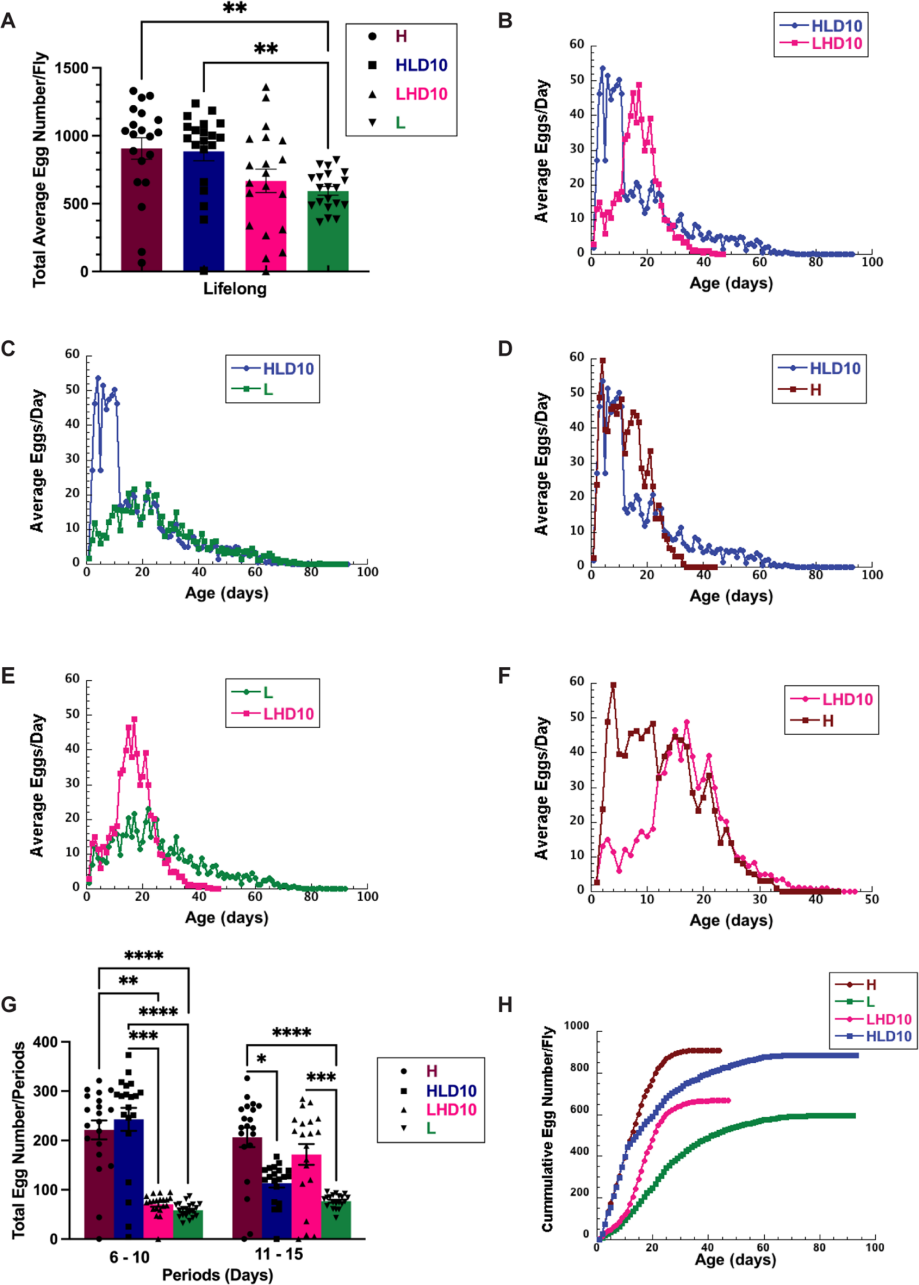
Calorie content of diet affects female life-long egg-laying patterns. **A** Total average lifelong egg number produced by female *CS* flies kept on a high-calorie diet (brown) or low-calorie diet (green), and flies shifted from a low- to high- (magenta) or from a high- to a low-calorie diet (blue) on day 10. **B** Comparison of average egg production of female flies shifted from a low (L)- to a high-calorie diet (H) or a high- to low-calorie diet at 10 days during whole life. **C** Comparisons of lifelong average daily egg production for female kept on a low (green) calorie diet to eggs produced by females shifted at 10 days from a high- to a low-calorie diet on day 10 (blue). **D** Comparisons of lifelong average daily egg production for females kept on a high-calorie diet (brown) to eggs produced by females shifted at 10 days from a high- to a low-calorie diet (blue). **E** Comparisons of lifelong average daily egg production for females kept on a low-calorie diet (green) to eggs produced by females shifted at 10 days from a low- to a high-calorie diet (magenta). **F** Comparisons of lifelong average daily egg production for females kept on a high-calorie diet (brown) to eggs produced by females shifted at 10 days from a low- to a high-calorie diet (magenta). **G** Total average egg production during the period between 6–10 and 11–15 days produced by female *CS* flies kept on a high- (brown) or low-calorie diet (green), and flies shifted from a low- to a high- (magenta) or from a high- to a low-calorie diet (blue) on day 10. **H** Cumulative egg production in wild-type female *CS* flies kept on a high- (brown) or low-calorie diet (green), and flies shifted from a low- to a high- (magenta) or from a high- to a low-calorie diet (blue) on day 10. One to 15 days were analyzed separately using the Kruskal–Wallis test. Post-hoc analysis was conducted using Dunn’s test, correcting for multiple comparisons using GraphPad Prism 9.4.1, Results represent SEM. There were 20 female flies in each experiment. *p*: 0.033 (*), 0.002 (**), 0.0002 (***), < 0.0001 (****)

While diet has profound effects on the survivorship of male and female flies, females experience additional physiological changes due to the effects of diet on fecundity and egg production. Therefore, it is important to uncover how caloric shifts affect female egg production. Previous work from our group, and others, has demonstrated that female flies modify their egg production depending on diet [22, 26, 49]. In order to carefully profile how egg production is modified when flies undergo caloric shifts, we measured egg production of females flies on lifelong high- and low-calorie diets, and after shifting diets in a manner similar to our lifespan studies; flies were shifted from high- to low-calorie diets (and vice versa) at two time points, 10 and 50 days. Numbers of eggs laid by each group were monitored daily and compared to the number of eggs produced by females shifted to the opposite diet and to eggs laid by female flies kept on a lifelong, low- or high-calorie diet. As expected, flies on a lifelong, low-calorie diet produced fewer eggs than those on a high-calorie diet (Fig. 3A).

However, as with lifespan, flies were immediately responsive to dietary shifts, and would increase or decrease the number of eggs produced depending on diet (Fig. 3B–F). Flies that were shifted from a high- to a low-calorie diet at day 10 responded with a sudden drop in egg production to levels that matched flies on a lifelong, low-calorie diet (Fig. 3C). This was further illustrated by a similar total number of eggs laid from days 11–15 by females shifted from a high- to a low-calorie diet on day 10 compared to females kept on a constant low-calorie diet during this same period (L = 66, SEM = 3.0, HLD10 = 114, SEM = 9.0) (Fig. 3G). Likewise, when shifted from a low- to a high-calorie diet at day 10, the opposite was found to be true; flies responded by increasing the number of eggs produced (Fig. 3E–F). Again, flies switched from a low- to a high-calorie diet at day 10 produced similar numbers of eggs 5 days after shifting (days 11–15), to the number of eggs produced by females kept on a high-calorie diet from day 1 during the same period (H = 206, SEM = 19.8; LHD10 = 172, SEM = 21.1) (Fig. 3G, Table 3).

However, flies that were shifted from a low- to a high-calorie diet did not produce the same cumulative number of eggs across their lifespan as flies raised on a constant, high-calorie diet (Fig. 3H). Since flies increased egg production to levels that match those on a high-calorie diet immediately after shifting, this loss in cumulative production is likely due to the missed opportunity to produce a high number of eggs within the first 10 days of life, when females produce the highest number of eggs [47]. Intriguingly, flies that were shifted from a high- to a low-calorie diet at day 10 produced the same, cumulative number of eggs (~ 900) across their lifespan as flies raised on a constant, high-calorie diet (Fig. 3A, H). This was strikingly different from flies that were maintained on a low-calorie diet throughout their lifespan, which produced a much smaller number of eggs compared to flies on a high-calorie diet (~ 600 vs 900). This suggests that female flies can benefit from lifespan extension via CR without a sacrifice in lifelong fecundity, if dietary components or calories are provided early in life.

Surprisingly, female flies were still able to modify egg production in response to caloric shifts at day 50. When flies were shifted from a low- to a high-calorie diet at that time point, there was a small increase in the number of eggs produced (Supplemental Fig. 2A). Interestingly, a small but clear increase in egg production was noted when shifted to a high-calorie diet despite very few flies remaining alive (Supplemental Fig. 2B). For example, at day 58, approximately 5% of flies remained, but egg production maintained at about eight eggs per fly. This was starkly contrasted by flies switched to a low-calorie diet, where about 80% of flies remained alive, but very few eggs, if any, were being produced. *Ultimately this finding highlights the fact that both fly lifespan and fecundity remain responsive to dietary shifts even at an old age, and that energy is immediately allocated to or from egg production depending on diet.*

### Metabolic alterations mediate the response to calorie restriction

Considering the dramatic response in lifespan and fecundity exhibited after late-life caloric shifts in female flies, we wanted to capture the underlying metabolic adaptations that may mediate some of such responses. To do this, we measured levels of triglycerides, glucose, glycogen, trehalose, and proteins in flies shifted from high- to low-calorie diets (and vice versa) at two time points, 20 and 50 days.

**Fig. 4:**
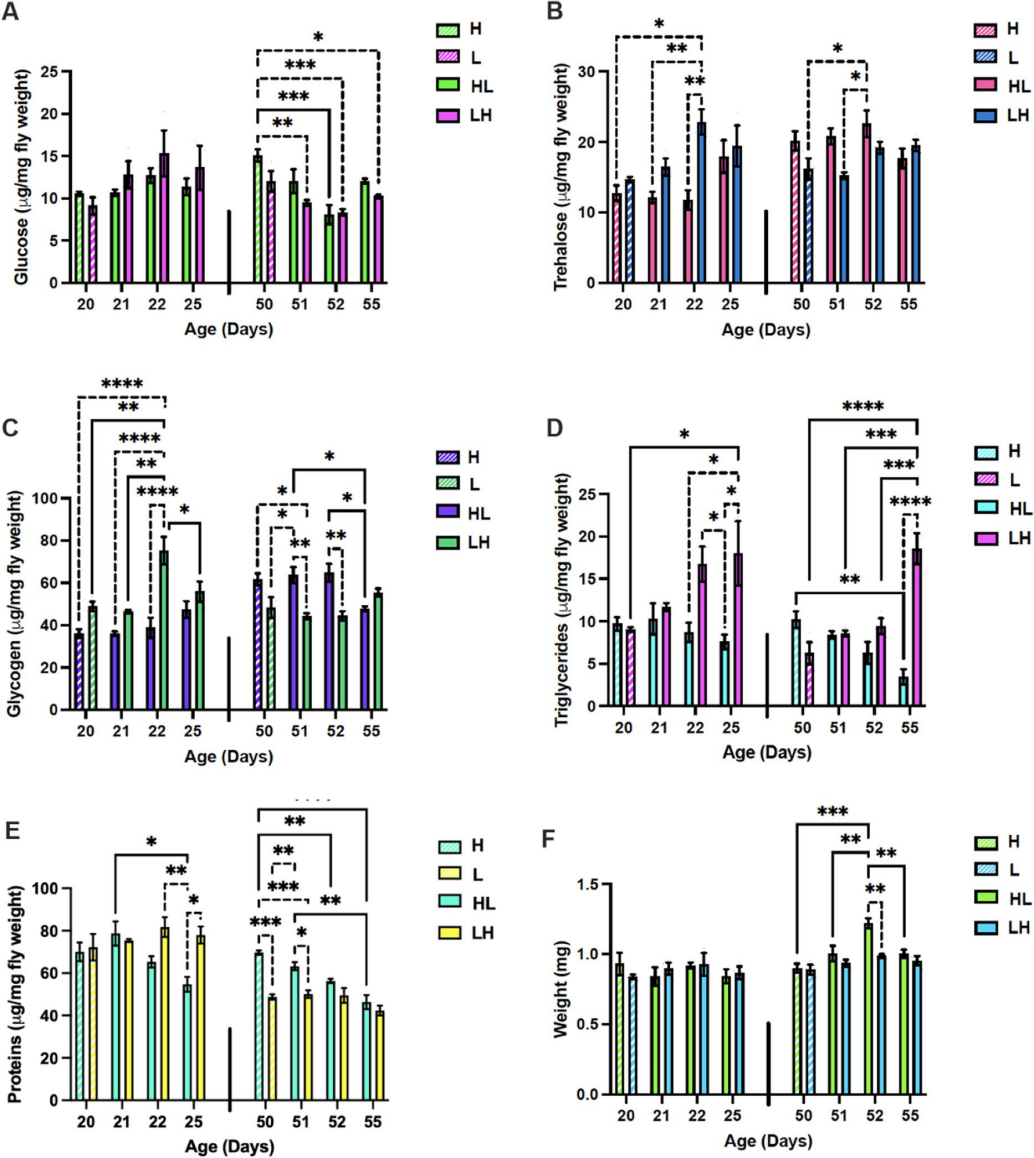
Effects of diet shifting on metabolism are age-dependent. Levels of glucose (**A**)**,** trehalose (**B**), glycogen (**C**), triglycerides (**D**), protein (**E**), and weight (**F**) in female flies on days 20 and 50 before shifting, and 1, 2, and 5 days after shifting from a high- to a low-calorie diet (HL) or L to H diet (LH). Weight is an average weight per fly, calculated from the three biological replicates (10 flies per replicate). Error bars represent SEM. Data from 20 and 50 days were analyzed separately using a two-way ANOVA. Post hoc analysis was conducted using Tukey’s test, correcting for multiple comparisons. Results represent means ± SE of three biological replicates (10 flies per replicate). *p*: 0.05 (*), 0.01 (**), 0.001 (***), < 0.0001 (****)

The biggest change was observed in triglycerides levels, which showed an increasing trend when switched from a low- to high-calorie diet at day 20, which was significantly different from baseline (day 20, low) at day 25 (Fig. 4D). This was exacerbated later in life, with triglyceride levels showing a significant increase each day the flies were on the high-calorie diet (Fig. 4D). The opposite was also true when flies were shifted from a high- to a low-calorie diet, levels of triglycerides began to drop. Again, this effect was more prominent in older flies, with a significant decline in triglyceride levels compared to baseline (day 50, low) (Fig. 4D). Increased lipid levels in diet affect the steroid hormone ecdysone production in the ovary, which regulates lipid accumulation in late-stage oocyte development, and promotes egg production. It also promotes whole body triglyceride and glycogen storage via the ecdysone receptor EcR signaling [50]. In addition, juvenile hormone, produced by *corpora allata*, stimulates vitellogenin production by the fat body, and vitellogenin sequestration by the oocytes [51]. *Together, this data suggests that female flies respond to dietary shifts by mobilizing or storing triglycerides, possibly to maintain tight regulation of glucose, trehalose, and glycogen levels, and egg production.*

Notably, flies appear to tightly regulate levels of glucose, glycogen, and trehalose at the 20-day time point, with levels remaining fairly stable upon both dietary switch regimes (Fig. 4A–C). While flies shifted from a low- to a high-calorie diet showed a significant increase in glycogen levels at day 22, the levels were not significantly different from baseline (day 20, low) at day 25, suggesting day 22 may be an anomaly (Fig. 4C). Similarly, when switched from a high- to a low-calorie diet at day 50, glycogen levels were significantly decreased by day 55, but there was not otherwise a clear, daily, decreasing trend with time on this dietary regime (Fig. 4C). However, levels of glucose did decrease in response to high-to-low shifts at day 50. Interestingly, protein levels did not change when females were shifted to high-calorie diet, and remained stable at every time point after shifting, most likely due to utilization for egg production (Fig. 4E). They were, however, responsive to low-calorie diets, at both time points (Fig. 4E). These results, paired with egg production, suggest flies may utilize the excess energy, particularly protein, associated with a high-calorie diet to prioritize egg production. Once calories become sparing and protein levels begin to drop, this may cause a shift in resource allocation away from egg production. This has been demonstrated previously, as protein is a key dietary component required for egg production [26, 29, 52].

## Discussion

Together, our results demonstrate that *CS* female flies can benefit from late-life caloric shifts with no associated fitness cost and reveal several of the metabolic adaptations that influence these events. Traditional evolutionary theory has suggested that caloric restriction is an environmental cue that signals to organisms the need to invest in somatic maintenance rather than reproduction [53]. Under this paradigm, organisms would increase survival in order to have the opportunity to reproduce under more favorable conditions. However, this theory has been contested in studies which have identified dietary restriction to be associated with a hidden cost, which renders flies less fecund and more susceptible to death when returned to normal dietary conditions [43]. However, this result may be dependent on strain, sex, diet, time of CR application, or more, as this was not the response identified in our and other studies [44].

It is quite possible that flies in our experiments were not calorie restricted enough to lead to maximal lifespan extension. However, lower caloric content of diet could have led to the hidden cost of CR that we did not observe. Thus, it is likely that flies in our experiments were not as calorically restricted as flies in other studies. Consistently, experiments in multiple organisms reinforce that the effects of CR depend on the age, sex, genetic background, calories in diet, time of CR diet application, and content of the diet [1, 6, 25].

Here, we find female flies immediately respond to calorie restriction with a decrease in mortality when applied at any age tested. Similar decreases in mortality rates were found in *CS* male flies after shifting at 20, 50, or 60 days [46]. As expected, female flies also decreased egg production; however, flies continued to produce eggs across their lifespan. Since flies live longer after shifting from a high- to a low-calorie diet, they ultimately produce the same cumulative number of eggs as flies on a high-calorie diet, albeit at a slower rate. Further, when switched to a high-calorie diet, younger flies did not experience an increase in mortality that was higher than flies raised on a constant, high-calorie diet. These flies also responded with an immediate increase in egg production, which rose to match levels of flies on a high-calorie diet. While female flies switched from a low- to a high-calorie diet did not produce the same, cumulative number of eggs across their entire lifespan, likely due to missing out on the initial burst of production that occurs within the first 10 days of life, starting on a low-calorie diet did not hinder their ability to increase egg production when switched to a high-calorie diet. Together, these results do not suggest a cost of calorie restriction in female flies; alternatively, they suggest the ability to optimize fitness of female flies through the utilization of dietary shifts. For example, if flies are raised on a constant, low-calorie diet, while they benefit from an increased lifespan, they produce significantly fewer eggs compared to flies on a constant, high-calorie diet. However, if flies are first raised on a high-calorie diet, and then switched to a low-calorie diet, they can benefit from an extended lifespan with no cost to cumulative reproduction.

While older female flies in our study displayed a transient increase in risk of dying when switched from a low- to a high-calorie diet, this increase did not endure as the flies aged. Additionally, flies in this dietary regime also responded with an increase in egg production. Similar to previous studies, this suggests that the increase in mortality when switched to a high-calorie diet may be due to the cost of reproduction, mating, and increased vulnerability in aged flies [44, 47, 49]. However, since flies in this group did not show a sustained increase in mortality after the switch occurred, and were able to begin producing similar numbers of eggs per fly as those in the high-calorie group, our results suggest that age may render female flies more vulnerable to the effects of reproduction and/or high-calorie diets, but not that there is a fitness cost to calorie restriction. This differs from previous observations in male flies, in which shifting from a low- to a high-calorie diet leads to a higher risk of dying compared to males aged on a lifelong, high-calorie diet, further emphasizing sex-dependent responses to diet shifting [46].

This study also uncovered some of metabolic adaptations that occur in response to dietary shifts. To gain a holistic understanding of the role of yeast and carbohydrates in diet, the Pletcher group analyzed how 25 different diet compositions impacted fly physiology, behavior, and lifespan [29]. They found increasing yeast to carbohydrate ratio increases fecundity, suppresses food consumption, and leads to lean flies, while increasing carbohydrates reduces fecundity, slightly increases food consumption, and leads to obese flies [29]. Because of the counteracting effects of dietary yeast and sucrose on food consumption levels, they found that flies kept on a diet with an equal amount of yeast and sugar, regardless of the total caloric amount, eat the same and do not gain weight. In our lab, we have previously examined food intake and found no change in volumetric food uptake in female flies at 10 and 20 days of age when aged on 0.5N, and 3.0N [22]. Similarly, Mair et al. reported that flies do not eat more volume of food to compensate for decreased nutrient content [54]. Based on our previous and published studies, female flies do not change food uptake after shifting. However, female flies shifted from a low- to a high-calorie diet consume a much higher number of calories present in the same volume of digested food. Once female flies were shifted to a high-calorie diet, the levels of triglycerides were significantly increased. Female flies that were shifted from a high- to a low-calorie diet experienced decreases in triglyceride levels. Similar reductions in triglyceride levels were found in human clinical studies in which healthy, obese individuals were subjected to calorie restriction. High-to-low shifting is associated with use of lipids, instead of glucose, as a major energy source. A similar metabolic adaptation was found in starved female flies when moved from a satiated conditions and was characterized by use of ketone bodies as a major source of energy instead of glucose [55]. Flies may also prioritize maintaining sugar levels, possibly mobilizing or storing triglycerides in order to achieve homeostatic regulation of carbohydrates. Flies seem to be increasingly vulnerable to the effects of shifting at an older age, as those shifted to a high-calorie diet showed profound increases in triglyceride levels compared to baseline (day 50, high). This increase occurs within the context of elevated mortality, corroborating the pathological effects of high triglycerides on flies [56]. It is tempting to think of this in the context of metabolic disease in humans, as high triglycerides are significantly associated with high risk of all-cause mortality [57]. Further, our data are consistent with previous reports and further emphasize the importance of protein levels in egg production, as flies shifted to low-calorie diets displayed decreasing levels of whole-body protein levels [29]. This occurred within the context of egg production decline, suggesting flies are limited by protein to produce eggs. Counter to this, flies may maintain homeostatic protein levels through an increase in egg production, investing excess protein gained on a high-calorie diet directly to egg production. Evidence has shown that limiting micronutrients, such as cholesterol, could also limit lifespan and egg production; therefore, we cannot discount the fact that flies on a high-calorie diet were limited by other nutrients due to the increase in egg production [58, 59]. Future experiments which utilize our dietary shift regime, along with addition of nutrients like cholesterol, would be interesting to evaluate whether this is a limiting factor in our experimental conditions. However, as described before, cholesterol supplementation is not sufficient to fully compensate for the costs of a high protein diet, and therefore, is not expected to abrogate our results [44, 58].

Ultimately, we find that female *CS* flies can benefit from CR, even when applied late in life. Flies can achieve substantial gains in lifespan despite very few flies remaining when shifted at the old age of 60 days. This gain in lifespan likely occurs by a decrease in risk of death, possibly mediated by metabolic adaptations (such as a decrease in triglycerides), a decrease in egg production, or a combination of other factors. Conversely, when switched to a high-calorie diet, flies experience an increase in mortality, which appears to be more costly when flies are shifted at later ages. Despite this increase in mortality, flies are at no higher a risk than flies on a lifelong, high-calorie diet, and have the capacity to produce the same number of eggs as their high-calorie counterparts. This suggests that there is no cost associated with calorie restriction, which may be a sex specific finding. Transcriptomic data obtained in male *CS* flies shifted from a low- to a high-calorie diet at 50 days provide some explanation for immediate increase in hazard rate. The authors hypothesized that male flies shifted to a high-calorie diet are suddenly exposed to increased energy levels that are immediately used for growth, proliferation, and storage of energy, all processes that are associated with release of reactive oxidative species. This hypothesis is supported by an increased number of differentially expressed genes in the glutathione family, cytochrome p450, and genes involved in growth and proliferation [46]. Finally, due to the dynamic ability of female flies to respond to dietary shifts, our findings imply that fly fitness could be optimized, maximizing both lifespan and fecundity utilizing caloric shifts.

### Supplementary Information

Below is the link to the electronic supplementary material.

Supplementary file1 (DOCX 999 KB)

## Data Availability

Data can be provided by the corresponding author upon request.
