# Supplementary material for: Extended lifespan in female Drosophila melanogaster through late-life calorie restriction
Source: GeroScience. 2024 Jul 2;46(5):4017–35. doi: 10.1007/s11357-024-01233-w (PMC11335708; doi:10.1007/s11357-024-01233-w)
Supplement: Supplementary file 1 — Supplementary file1 (DOCX 999 KB) [file 11357_2024_1233_MOESM1_ESM.docx]

**Supplemental Information**

**Extended lifespan in female *Drosophila melanogaster* through late-life calorie restriction**

**GeroScience**

**Michael Li^1^, Jacob Macro^1^, Billy J. Huggins^1^, Kali Meadows^1^, Dushyant Mishra^1^, Dominique Martin^1^, Kavitha Kannan^1^, Blanka Rogina^1,2,*^**

**^1^**Department of Genetics & Genome Sciences, School of Medicine, University of Connecticut Health, Farmington, CT, 06030, USA

**^2^**Institute for Systems Genomics, School of Medicine, University of Connecticut Health, Farmington, CT, 06030, USA

**^*^Corresponding author:** [**rogina@uchc.edu**](mailto:rogina@uchc.edu) **ORCID:** 0000-0003-1195-905X

**Supplemental Figure Legends**

**Fig. S1** **Shifting diets has immediate effects on female lifespan:** Survivorships between 50 and 85 (**A**) or 60 – 85 (**B**) of female flies shifted from a high (H) to a low (L) calorie diet at day 50 (HLD50) (**A**) or day 60 (HDL60) (**B**) or from L to H diet on day 50 (LHD50) (A) or day 60 (LHD60) (B). Number of flies: Number of flies: L=439, H=402, HLD50=217, HLD60=211, LHD50=226, LHD60=218.

Survivorships curves were analyzed by long-rank test JMP16 program.

**Fig. S2 Shifting flies to diet with different calorie content affect female egg production:** Average daily egg production (**A**) and survivorships (**B**) between 50 and 60 days of female *CS* flies shifted from a low to a high calorie diet (LHD50) or from a high to a low (HLD50) calorie diet at day 50. Number of flies: HLD50=20, LHD50=20.

**Supplemental Figure 1**

**Supplemental Figure 2**

**Supplemental Tables**

**Supplemental Table 1: Effects of shifting *Canton-S* female flies from a high (H) to a low (L) calorie diet on longevity compared to longevity of flies on lifelong high calorie diet.**

| **Food** | **Time on L diet** | **N**  **(n censored)** | **Mean LS (% change)** | ***X*^2^** | **p** | **Maximal LS (% change)** |
| --- | --- | --- | --- | --- | --- | --- |
| H | Lifelong | 227 (3) | 46.1 (-80.7) |  | <0.0001* | 70.7 (-45.1) |
| L | Lifelong | 194 (1) | 83.3 |  |  | 102.5 |
| H | Lifelong | 227 (3) | 46.1 (-74.2) | 329.1587 | <0.0001* | 70.7 (-48.1) |
| HLD10 | D10 | 219 (4) | 80.3 |  |  | 104.7 |
| H | Lifelong | 227 (3) | 46.1 (-57.9) | 243.4023 | <0.0001* | 70.7 (-45.8) |
| HLD20 | D20 | 225 (16) | 72.8 |  |  | 103.0 |
| H | Lifelong | 227 (3) | 46.1 (-38.2) | 113.99962 | <0.0001* | 70.7 (-44.9) |
| HLD30 | D30 | 211 (6) | 63.7 |  |  | 102.4 |
| H | Lifelong | 227 (3) | 46.1 (-43.8) | 109.4052 | <0.0001* | 70.7 (-47.5) |
| HLD40 | D40 | 222 (5) | 66.3 |  |  | 104.2 |
| H | Lifelong | 227 (3) | 46.1 (-12.8) | 14.3933 | <0.0001* | 70.7 (-40) |
| HLD50 | D50 | 229 (0) | 52.0 |  |  | 98.6 |

L=Low calorie diet

H=High calorie diet

HLD10= flies shifted from a high to low calorie diet at day 10

HLD20= flies shifted from a high to low calorie diet at day 20

HLD30= flies shifted from a high to low calorie diet at day 30

HLD40= flies shifted from a high to low calorie diet at day 40

HLD50= flies shifted from a high to low calorie diet at day 50

N = Number of flies in experiment used for calculating mean and maximal lifespan

n censored = number of flies that died between 0 – 10 days, and are not included in listed N or included in calculation of mean and maximal lifespan. The total number of flies in experiments on Day 0 is N plus n flies.

*Statistically significant

**Supplemental Table 2: Effects of shifting *Canton-S* female flies from a low (L) to a high (H) calorie diet on longevity in comparison to longevity of flies on lifelong low calorie diet.**

| **Food** | **Time on L diet** | **N**  **(n censored)** | **Mean LS (% change)** | ***X*^2^** | **p** | **Maximal LS (% change)** |
| --- | --- | --- | --- | --- | --- | --- |
| L | Lifelong | 194 (1) | 83.3 (44.6) | 359.1320 | <0.0001* | 102.5 (31.1) |
| H | Lifelong | 227 (3) | 46.1 |  |  | 70.7 |
| L | Lifelong | 194 (1) | 83.3 (40.6) | 258.5166 | <0.0001* | 102.5 (22.7) |
| LHD10 | D10 | 231 (1) | 49.5 |  |  | 79.2 |
| L | Lifelong | 194 (1) | 83.3 (40.2) | 286.1931 | <0.0001* | 102.5 (23.7) |
| LHD20 | D20 | 242 (11) | 49.8 |  |  | 78.2 |
| L | Lifelong | 194 (1) | 83.3 (34.8) | 248.8852 | <0.0001* | 102.5 (20.4) |
| LHD30 | D30 | 199 (8) | 54.3 |  |  | 81.6 |
| L | Lifelong | 194 (1) | 83.3 (36.5) | 313.1890 | <0.0001* | 102.5 (35.1) |
| LHD40 | D40 | 228 (3) | 52.9 |  |  | 66.5 |
| L | Lifelong | 194 (1) | 83.3 (32) | 325.2695 | <0.0001* | 102.5 (27.8) |
| LHD50 | D50 | 224 (4) | 56.2 |  |  | 74.0 |

L=Low calorie diet

H=High calorie diet

LHD10= flies shifted from a high to low calorie diet at day 10

LHD20= flies shifted from a high to low calorie diet at day 20

LHD30= flies shifted from a high to low calorie diet at day 30

LHD40= flies shifted from a high to low calorie diet at day 40

LHD50= flies shifted from a high to low calorie diet at day 50

N = Number of flies in experiment used for calculating mean and maximal lifespan

n censored = number of flies that died between 0 – 10 days, and are not included in listed N or included in calculation of mean and maximal lifespan. The total number of flies in experiments on Day 0 is N plus n flies).

*Statistically significant

**Table 3A: Hazard ratio (HR) of *Canton S* (*CS*) female flies shifted at 20 days from a high (H) to a Low (L) calorie diet (HL) or vice-versa (LH) and compared to hazard ratio of flies kept on a constant high (H) or low (L) calorie diet.**

| Period  (days) | HR H&HLD20 | p | HR H&LHD20 | p | HR L&HLD20 | p | HR L&LHD20 | p |
| --- | --- | --- | --- | --- | --- | --- | --- | --- |
| 20-30 | 0.42 | 0.07 | 0.96 | 0.88 | 0.66 | 0.47 | 1.31 | 0.5 |
| 30-40 | 0.08 | 0.01** | 0.86 | 0.46 | 0.24 | 0.07 | 1.36 | 0.41 |
| 40-50 | 0.43 | 0.05* | 1.05 | 0.76 | 0.57 | 0.32 | 1.39 | 0.39 |
| 50-60 | 0.49 | 0.1 | 1.1 | 0.61 | 0.82 | 0.64 | 1.03 | 0.92 |
| 60-70 | 0.41 | 0.01** | 0.76 | 0.22 | 1.18 | 0.67 | 0.8 | 0.43 |

*Statistically significant

**Table 3B: Hazard ratio (HR) of *Canton S* (*CS*) female flies shifted at 50 days from a high (H) to a Low (L) calorie diet (HL) or vice-versa (LH) and compared to hazard ratio of flies kept on a constant high (H) or low (L) calorie diet.**

|  | | |  |
| --- | --- | --- | --- |
|  |  |  | |

| Period (days) | HR H&HLD50 | p | HR H&LHD50 | p | HR L&HLD50 | p | HR  L&LHD50 | p |
| --- | --- | --- | --- | --- | --- | --- | --- | --- |
| 50-60 | 1 | 0.99 | 1.32 | 0.05* | 0.66 | 0.28 | 1.56 | 0.11 |
| 60-70 | 0.28 | 0.01** | 0.62 | 0.01** | 0.18 | 0.05* | 1.12 | 0.67 |

*Statistically significant

**Table 3C: Hazard ratio (HR) of *Canton S* (*CS)* female flies shifted at 60 days from a high (H) to a Low (L) calorie diet (HL) or vice-versa (LH) and compared to hazard ratio of flies kept on a constant high (H) or low (L) calorie diet.**

|  |
| --- |

| Period (days) | HR H&HLD60 | p | HR H&LHD60 | p | HR L&HLD60 | p | HR L&LHD60 | p |
| --- | --- | --- | --- | --- | --- | --- | --- | --- |
| 60-70 | 0.43 | 0.01** | 1.21 | 0.2 | 0.66 | 0.28 | 1.64 | 0.05* |

*Statistically significant
